# Editorial: Autophagy and lung cancer therapy: targeted drug development and new emerging technology

**DOI:** 10.3389/fphar.2025.1599548

**Published:** 2025-04-16

**Authors:** Ming Zhang, Yu Wang, Chunhui Yang, Xin You, Yafei Feng, Xianglin Yang, Qi Wang

**Affiliations:** ^1^ Translational Research Center for Lung Cancer, The Second Hospital, Dalian Medical University, Dalian, Liaoning, China; ^2^ Department of Pharmacy, The Second Hospital, Dalian Medical University, Dalian, Liaoning, China; ^3^ Department of Cardiology, The Second Hospital, Dalian Medical University, Dalian, Liaoning, China; ^4^ Department of Clinical Laboratory, The Second Hospital, Dalian Medical University, Dalian, Liaoning, China; ^5^ Department of Respiratory Medicine, The Second Hospital, Dalian Medical University, Dalian, Liaoning, China; ^6^ Department of Discipline Construction and Scientific Research Management, The Second Hospital of Dalian Medical University, Dalian, Liaoning, China

**Keywords:** autophagy, lung cancer, targeted therapy, precision medicine, mitophagy

## Introduction

Lung cancer remains one of the most aggressive malignancies worldwide (Bade and Dela Cruz, 2020). Despite significant advances in traditional therapeutic approaches—including chemotherapy, radiation, and surgical interventions—the emergence of resistance mechanisms continues to limit their effectiveness (Shien et al., 2017). The advent of precision medicine has shifted focus toward targeted therapies capable of selectively affecting cancer cells while sparing normal tissues, thereby offering enhanced efficacy with reduced toxicity compared to conventional chemotherapeutic agents (Ruiz-Cordero and Devine, 2020).

In recent years, autophagy—a fundamental cellular process that mediates the degradation and recycling of cellular components—has emerged as a crucial player in cancer biology, including lung cancer (Wang et al., 2022). This self-eating mechanism serves as a double-edged sword in cancer: it can promote tumor cell survival under stress conditions or, conversely, induce cell death when hyperactivated (Zhang et al., 2023). The complex role of autophagy in tumorigenesis, progression, metastasis, and treatment response has positioned it as a promising target for therapeutic intervention in lung cancer (Biswas et al., 2024).

This Research Topic, “Autophagy and Lung Cancer Therapy: Targeted Drug Development and New Emerging Technology,” aimed to provide a comprehensive overview of recent advances in autophagy-related research in lung cancer. The Research Topic encompasses studies identifying novel molecular targets, developing innovative diagnostic technologies, and exploring therapeutic strategies targeting autophagy pathways. The contributions to this Research Topic highlight the multifaceted aspects of autophagy in lung cancer through original research articles, case reports, and systematic reviews.

## Overview of contributions

The Research Topic begins with a comprehensive review by Zhang et al. This article delves into mitophagy—a specialized form of autophagy responsible for the selective degradation of mitochondria—and its critical involvement in lung cancer initiation, progression, metastasis, and treatment response. The authors meticulously detail the regulatory landscape of mitophagy, highlighting how aberrations in this process contribute to the tumor microenvironment. Furthermore, they explore the potential of mitophagy modulators, both inhibitors and activators, as therapeutic agents while emphasizing the importance of targeting specificity to minimize damage to healthy cells. The review also discusses mitophagy-related molecular components as potential biomarkers for enhancing diagnostic accuracy, prognostic assessment, and prediction of therapeutic responses.

In the realm of clinical perspectives, Sun et al. present a rare case report. This study documents a severe case of toxic epidermal necrolysis (TEN) in a lung adenocarcinoma patient following Camrelizumab monotherapy. The authors describe the successful management approach using high-dose corticosteroids and intravenous immunoglobulin therapy. Through a comprehensive literature review, they provide insights into the characteristics and treatment options for immune-related TEN, offering valuable guidance for the safe application of immune checkpoint inhibitors in clinical practice.

A research article by Wang et al. investigates the role of Nicotinamide N-methyltransferase (NNMT) in drug sensitivity and resistance in non-small cell lung cancer (NSCLC). Through proteomic analysis and gene expression studies, the authors demonstrate that NNMT knockdown enhances sensitivity to osimertinib by modulating autophagy pathways. Their development of a predictive model based on NNMT-associated gene expression offers a potential tool for forecasting patient survival outcomes. This work underscores the significance of NNMT as both a therapeutic target for overcoming chemoresistance and a predictive marker for clinical outcomes in lung cancer.

Another noteworthy case report by Xu et al. presents the first documented case of lorlatinib efficacy in treating advanced high-grade pulmonary mucoepidermoid carcinoma (PMEC) harboring the EML4-ALK fusion V2 mutation. This case highlights the importance of comprehensive molecular profiling even in rare lung cancer subtypes and illustrates the potential benefits of targeted therapies in such contexts.

Liu et al. contribute a systematic bibliometric analysis. This comprehensive review maps the knowledge structure and evolutionary trends in autophagy-lung cancer research over the past decade. The authors identify five major research clusters. This analysis provides valuable insights into research trends and highlights emerging frontiers, including cell proliferation, migration, epithelial-mesenchymal transition, and the tumor microenvironment as promising areas for future investigation.

Finally, the Research Topic includes an economic evaluation by Dai et al. This study employs a partitioned survival model to assess the cost-effectiveness of iruplinalkib, a second-generation ALK tyrosine kinase inhibitor developed in China, compared to alectinib in ALK-positive crizotinib-resistant advanced NSCLC. The authors demonstrate that iruplinalkib offers a favorable cost-effectiveness profile in the Chinese healthcare setting, providing important pharmacoeconomic data to inform clinical decision-making and healthcare resource allocation.

## Future perspectives

The Research Topic of papers in this Research Topic illustrates the complexity and multidimensional nature of autophagy in lung cancer biology and therapeutics. Several key themes emerge that will likely shape future research directions:

1. Precision Medicine Approaches: The identification of autophagy-related biomarkers and molecular targets offers opportunities for personalized therapeutic strategies tailored to specific patient populations.
2. Novel Drug Development: The exploration of compounds that modulate autophagy, whether through inhibition or activation, represents a promising avenue for overcoming treatment resistance in lung cancer.
3. Combination Therapies: Understanding how autophagy influences response to existing treatments suggests potential for rational combination strategies that enhance therapeutic efficacy.
4. Immune-Autophagy Interactions: The relationship between autophagy and immune responses within the tumor microenvironment merits further investigation, particularly in the context of immunotherapy resistance mechanisms.
5. Economic Considerations: As illustrated by the cost-effectiveness analysis included in this Research Topic, the economic impact of novel therapies must be evaluated alongside clinical efficacy to ensure sustainable implementation in healthcare systems.

## Conclusion

This Research Topic provides a multifaceted examination of autophagy in lung cancer, spanning from basic cellular mechanisms to clinical applications and economic assessments. The diverse contributions highlight both the progress made in understanding autophagy-related processes in lung cancer and the challenges that remain in translating this knowledge into effective therapeutic strategies.

By bringing together these varying perspectives, we hope to stimulate further research into autophagy-targeted approaches for lung cancer therapy and accelerate the development of innovative diagnostic and therapeutic tools. As our understanding of autophagy in cancer biology continues to evolve, so too will our ability to harness this fundamental cellular process for improving outcomes in patients with lung cancer.
